# Is there a prognostic difference among stage I lung adenocarcinoma patients with different *BRAF* ‐mutation status?

**DOI:** 10.1111/1759-7714.15248

**Published:** 2024-02-16

**Authors:** Shang‐Shang Ma, Rang‐Rang Wang, Qiao Peng, Yu'e Liu, Jia‐Yi Qian, Ming‐Jun Li, Kun Li, Zhi‐Ye Huang, Lei‐Lei Wu, Dong Xie

**Affiliations:** ^1^ Department of Thoracic Surgery, Shanghai Pulmonary Hospital, School of Medicine Tongji University Shanghai P. R. China; ^2^ School of Medicine Tongji University Shanghai P. R. China

**Keywords:** BRAF V600E mutations, lung adenocarcinoma, overlap weighting, prognosis

## Abstract

**Background:**

The data of the prognostic role of V‐Raf murine sarcoma viral oncogene homolog B1 (*BRAF*) mutations in early‐stage lung adenocarcinoma (LUAD) patients is scarce. This study aimed to investigate the proportion, clinicopathological features, and prognostic significance of patients with stage I LUAD carrying *BRAF* mutations.

**Methods:**

We collected 431 patients with pathological stage I LUAD from cBioPortal for Cancer Genomics and 1604 LUAD patients tested for BRAF V600E and epidermal growth factor receptor (EGFR) mutations from Shanghai Pulmonary Hospital. Survival curves were drawn by the Kaplan–Meier method and compared by log‐rank test. Cox proportional hazard models, propensity‐score matching (PSM), and overlap weighting (OW) were performed in this study. The primary endpoint was recurrence‐free survival (RFS).

**Results:**

The proportion of *BRAF* mutations was estimated at 5.6% in a Caucasian cohort. *BRAF* V600E mutations were detected in six (1.4%) patients in Caucasian populations and 16 (1.0%) patients in Chinese populations. Two *BRAF* V600E‐mutant patients were detected to have concurrent *EGFR* mutations, one for 19‐del and one for L858R. For pathological stage I LUAD patients, *BRAF* mutations were not significantly associated with worse RFS than wild‐type *BRAF* patients (HR = 1.111; *p* = 0.885). After PSM and OW, similar results were presented (HR = 1.352; *p* = 0.742 and HR = 1.246; *p* = 0.764, respectively). *BRAF* V600E mutation status also lacked predictive significance for RFS (HR, 1.844; *p* = 0.226; HR = 1.144; *p* = 0.831 and HR = 1.466; *p* = 0.450, respectively).

**Conclusions:**

In this study, we demonstrated that BRAF status may not be capable of predicting prognosis in stage I LUAD patients. There is a need for more data to validate our findings.

## Graphical abstract

A total of 431 patients from cBioPortal for Cancer Genomics and 1604 patients from Shanghai Pulmonary Hospital were included in order to investigate the proportion, clinicopathological features, and prognostic significance of patients with stage I LUAD carrying *BRAF* mutations. Overall, *BRAF* V600E mutations were detected in six (1.4%) patients in Caucasian populations and 16 (1.0%) patients in Chinese populations. In addition, we demonstrated that BRAF status may not be capable of predicting prognosis in stage I LUAD patients. There is a need for more data to validate our findings.

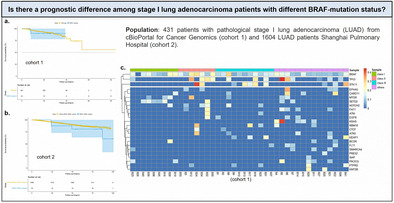

## INTRODUCTION

Lung cancer remains the leading cause of cancer death.^1^ Among lung cancer, non‐small cell lung cancer (NSCLC) accounts for about 85% of cases and can be divided into two major subtypes: lung adenocarcinoma (LUAD) and nonadenocarcinoma. Recently, the molecular landscape of NSCLC has been profoundly interrogated, benefiting from the advances of high‐throughput sequencing technologies.^2^ The discovery of mutations and rearrangements including epidermal growth factor receptor (EGFR) mutations, anaplastic lymphoma kinase (ALK), and ROS proto‐oncogene 1 (ROS1) rearrangements has led to the development of specific targeted agents and dramatically altered the therapeutic landscape, particularly regarding adenocarcinomas.^3^ Until now, other potential targets such as *BRAF* mutations have been probed and relevant efficacy data are emerging.

V‐Raf murine sarcoma viral oncogene homolog B1 (BRAF), a serine/threonine protein kinase, together with V‐Raf murine sarcoma 3611 viral oncogene homolog 1 (ARAF) and V‐Raf‐1 murine leukemia viral oncogene homolog 1 (CRAF), belongs to the RAF family. Physiologically, BRAF proteins are activated via rat sarcoma viral oncogene homolog (RAS) phosphorylation and then in turn phosphorylate mitogen‐activated protein kinase kinase 1/2 (MEK1/2) and subsequently mitogen‐activated protein kinase 1/2 (ERK1/2).^4^ Through the MEK–ERK pathway, BRAF plays a key role in regulating cell proliferation, differentiation and survival.^5^ Consequently, the mutation of *BRAF* may frustrate the negative feedback mechanism of the pathway and constitutively activate this signaling. *BRAF* mutations have been identified in about 3%–8% of all cancers, with a less frequent incidence in lung cancer.^6^ The majority of *BRAF* mutations occur in exon 15, corresponding to the substitution from valine to glutamate at codon 600 (V600E).

Pivotal phase II trials have investigated the efficacy of dual BRAF/MEK inhibition (dabrafenib in combination with trametinib) in patients harboring *BRAF* V600E mutations. The impressive results prompted the Food and Drug Administration (FDA) and European Medicines Agency (EMA) rapid approval of the regimen in clinical setting.^7^, ^8^ The encouraging outcomes urged us to further explore the *BRAF* mutations. Nevertheless, limited by the small number of patients, the impact of *BRAF* mutations on prognosis in LUAD remains unclear.^3^, ^9^, ^10^ In this study, therefore, we evaluated the data of 1604 stage I LUAD patients from the Shanghai Pulmonary Hospital and 431 patients from cBioPortal for Cancer Genomics, assessed the impact of BRAF mutation on prognosis and identified the clinical features of patients harboring *BRAF* mutations.

## METHODS

### Patient selection

**FIGURE 1:**
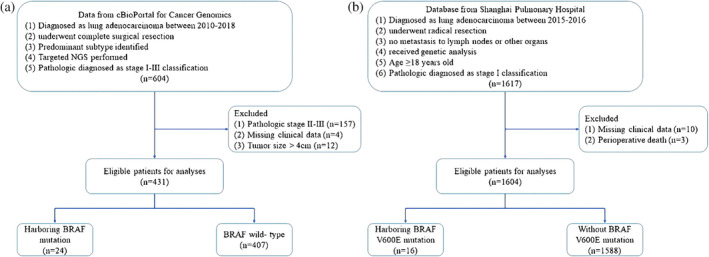
The flow chart of patient selection from cBioPortal for Cancer Genomics (a) and Shanghai Pulmonary Hospital (b).

This study was approved by the Ethics Committee of Shanghai Pulmonary Hospital (approval no. K23‐208). Public data were accessed via cBioPortal for Cancer Genomics (https://www.cbioportal.org/datasets). We obtained the data from the cohort from the database of Memorial Sloan Kettering‐Integrated Mutation Profiling of Actionable Cancer Targets (MSK‐IMPACT).^11^ This cohort included 604 LUAD patients who received an operation without neoadjuvant therapy. In addition, those patients were confirmed as having pathological stage I–III disease, and their predominant histological subtypes were identified and recorded. The flow chart of case selection is presented in Figure 1a. Finally, 431 patients with stage I LUAD from the database of MSK‐IMPACT were included in cohort 1.

We also collected data from the Shanghai Pulmonary Hospital from 2015 to 2016. Patients were included in this study if they met all of the following criteria: (1) a clear pathological diagnosis of LUAD, (2) had undergone radical resection, (3) had no metastasis to lymph nodes or other organs, and (4) received genetic analysis (amplification refractory mutation system [ARMS] polymerase chain reaction [PCR]). Patients who met any of the following conditions were excluded from this study: (1) age < 18 years, (2) perioperative death (died within 1 month after operation), (3) with other malignant tumors, and (4) had missing clinical data. Preoperative staging was performed and strictly followed the guidelines. The pathological tumor‐node‐metastasis (TNM) stage was identified according to the eighth edition classification system. Detailed information concerning patient selection is presented in Figure 1b. A total of 1604 eligible patients were included (cohort 2).

### Follow‐up information

In cohort 1, the median follow‐up time was 30.1 months, estimated using the reverse Kaplan–Meier method. As for cohort 2, the follow‐up information was updated in May 2023. The relevant information was obtained through telephone calls or medical records. The median follow‐up interval was 70.2 months (from 1 to 100 months). The primary observation endpoint of the present study was recurrence‐free survival (RFS). RFS was calculated from the date of surgery to the date of the first recurrence or last observation. Recurrence was confirmed by tissue biopsy or detailed examination, which included chest computed tomography, brain magnetic resonance imaging, radionuclide bone imaging, or positron emission tomography‐computed tomography.

### Statistical analysis

Pearson's Chi‐square and Fisher's exact tests were used to assess the proportions of categorical outcomes through the software SPSS 26.0 (IBM SPSS Inc.). Independent prognostic predictors were identified through the univariable Cox proportional hazards models and the results are presented as hazard ratio (HR) and 95% confidence interval (CI). RFS was calculated using the Kaplan–Meier method, and the differences were compared by log‐rank test. All survival curves were constructed by R version 4.1.1 software (https://www.r-project.org/). Statistical analyses were based on the two‐tailed hypothesis and statistical significance was considered as *p* < 0.05.

To minimize the bias caused by the different backgrounds of patients, we used propensity‐score matching (PSM) and overlap weighting (OW) in this study.^12^, ^13^, ^14^ OW is regarded as a propensity‐score method that attempts to mimic attributes of randomized clinical trials.^15^ In addition, OW assigns weights to each patient that are proportional to the probability of patients pertaining to the opposite group, thus can be as efficient as randomization if no adjustment is needed.^16^ In cohort 1, covariates including gender, age, smoking history, tumor size, and predominant histological pattern were balanced through PSM and OW. In cohort 2, the balanced covariates included gender, age, smoking history, tumor size, predominant histological pattern, visceral pleural invasion (VPI), lymphovascular invasion (LVI), spread through air spaces (STAS), and extent of surgery.

## RESULTS

### Patient characteristics

The baseline characteristics of cohort 1 are presented in Table 1. A total of 24 patients were found to harbor *BRAF* mutations, of which six (25.0%) tumors in patients were *BRAF* V600 mutations, and 18 (75.0%) were non‐V600 *BRAF* mutations. In the *BRAF*‐mutant subgroup, females outnumbered males, constituting 62.5% of the patients. A total of 20 (83.3%) patients had a smoking history. Predominant patterns were as follows: acinar/papillary 13 (54.2%), micropapillary/solid five (20.8%), and lepidic six (25.0%). The estimated *BRAF* mutation rate in LUAD was 5.6% and the proportion of *BRAF* V600 mutations was around 1.4%.

**TABLE 1:** The baseline characteristics of adenocarcinoma patients in cohort 1.

Variables	BRAF‐mutant		*p‐*value	SMD
	No	Yes		
Gender			0.694	0.128
Male	128 (31.4%)	9 (37.5%)		
Female	279 (68.6%)	15 (62.5%)		
Age at surgery, years			0.002	0.836
≤65	170 (41.8%)	2 (8.3%)		
>65	237 (58.2%)	22 (91.7%)		
Smoking history			0.857	0.096
No	83 (20.4%)	4 (16.7%)		
Yes	324 (79.6%)	20 (83.3%)		
Predominant pattern			0.225	0.349
Lepidic	67 (16.5%)	6 (25.0%)		
Acinar/papillary	288 (70.8%)	13 (54.2%)		
Micropapillary/solid	52 (12.8%)	5 (20.8%)		
Tumor size, cm			0.227	0.482
<1	18 (4.4%)	2 (8.3%)		
1–2	224 (55.0%)	17 (70.8%)		
2–3	125 (30.7%)	3 (12.5%)		
3–4	40 (9.8%)	2 (8.3%)		
BRAF status			<0.001	0.816
Wild‐type	407 (100.0%)	0 (0.0%)		
V600 mutation	0 (0.0%)	6 (25.0%)		
Non‐V600	0 (0.0%)	18 (75.0%)		

Abbreviation: SMD, standardized mean difference.

In cohort 2, a total of 1604 patients with surgically resected stage I LUAD were included. The baseline characteristics of patients are presented in Table S1. In the V600E‐mutant subgroup, females were equal to males, constituting 50.0% of the patients. A total of 15 (93.8%) patients were nonsmokers and only one patient had a smoking history. One patient was stage IA1, seven patients were stage IA2, five patients were stage IA3, and three patients were stage IB. We also observed two V600E‐mutant patients concurrent with *EGFR* mutations, one for 19‐del and the other for L858R. *BRAF* V600E mutations were harbored in a total of 16 patients, indicating a proportion of 1.0% for *BRAF* V600E mutation.

### Survival analyses

**FIGURE 2:**
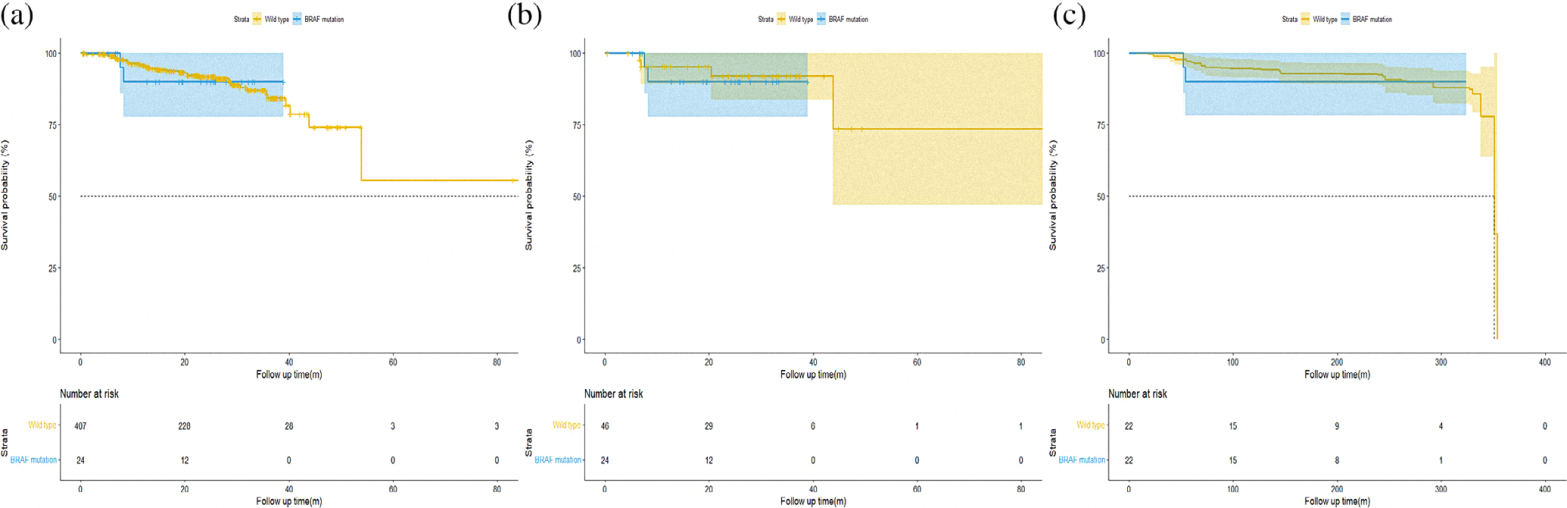
Kaplan–Meier survival curves for patients with lung adenocarcinoma in cohort 1. recurrence‐free survival before (a) matching, (b) after propensity‐score matching (PSM), and (c) after overlap weighting (OW).

We first analyzed the survival outcomes by Kaplan–Meier methods and Cox regression to evaluate the prognostic significance between *BRAF* mutant and wild‐type patients in cohort 1. As shown in Figure 2a, the survival curve revealed that the difference in RFS between patients with pathological stage I LUAD harboring *BRAF* mutations and *BRAF* wild‐type was not significant (median survival time not reached; HR, 1.111; *p* = 0.885). The joint effect of gender, age, smoking history, surgery type, predominant pattern, tumor size, VPI, LVI, STAS, and adjuvant therapy (ACT) was examined utilizing stepwise Cox regression analysis. The results of the univariable analysis are shown in Table 2. In the univariable analysis, *BRAF* mutation status was excluded in the equation as the independent factor to predict RFS (V600, HR, 3.403; *p* = 0.230; non‐V600, HR, 0.665; *p* = 0.688). To reduce potential bias and further confirm the results, PSM and OW were conducted. After adjustment, the results of PSM and OW revealed that patients with *BRAF* mutations had a similar trend of survival outcome with *BRAF* wild‐type patients (Figure 2b,c, adjusted HR, 1.352; *p* = 0.742 and HR, 1.246; *p* = 0.764, respectively).

**TABLE 2:** Univariable survival analyses of patients in cohort 1 for RFS.

Variables	Univariable analysis		
	HR	95% Cl	*p‐*value
Gender			
Male	1		
Female	0.985	0.501–1.939	0.965
Age at surgery, years			
≤65	1		
>65	1.083	0.570–2.056	0.808
Smoking history			
No	1		
Yes	1.058	0.485–2.308	0.887
Predominant pattern			
Lepidic	1		
Acinar/papillary	3.725	0.879–15.774	0.074
Micropapillary/solid	11.888	2.696–52.434	<0.001
Tumor size, cm			
<1	1		
1–2	0.805	0.183–3.545	0.774
2–3	1.781	0.400–7.918	0.448
3–4	1.050	0.191–5.768	0.955
Class			
Wild‐type	1		
V600	3.403	0.461–25.123	0.230
Non‐V600	0.665	0.091–4.863	0.688

Abbreviations: CI, confidence interval; HR, hazard ratio; RFS, recurrence‐free survival.

**FIGURE 3:**
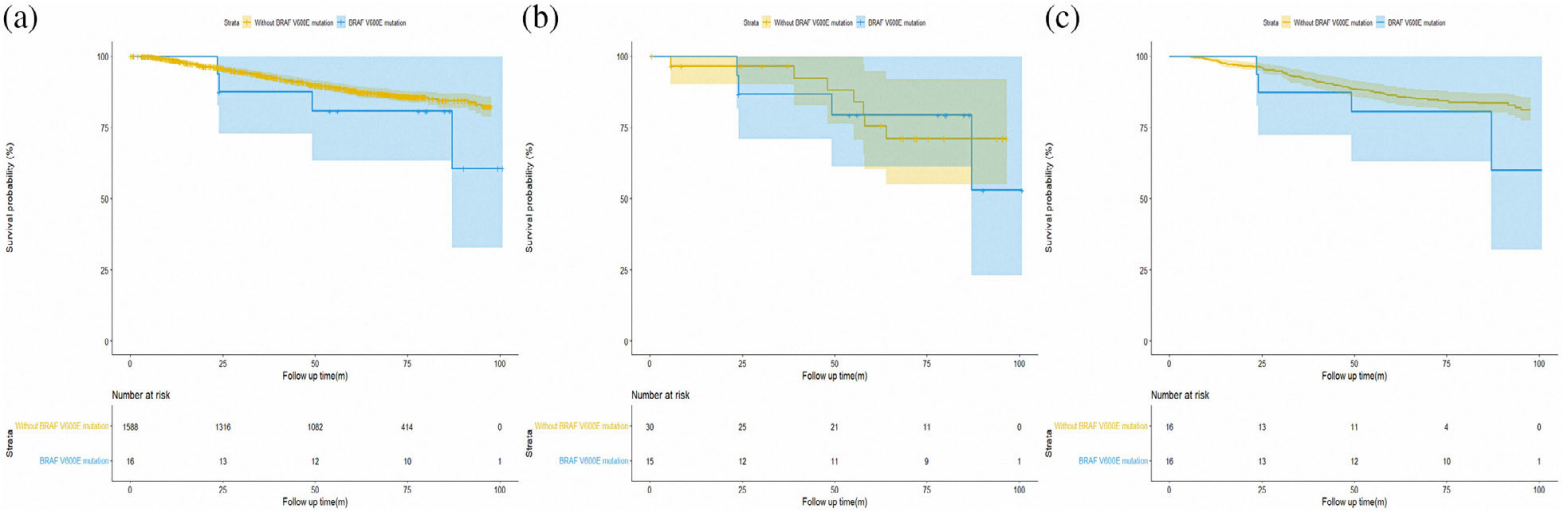
Kaplan–Meier survival curses for patients with lung adenocarcinoma in cohort 2. Recurrence‐free survival before (a) matching, (b) after propensity‐score matching (PSM), and (c) after overlap weighting (OW).

To further explore the prognostic role of *BRAF* V600E mutation, we then analyzed the data of cohort 2. As shown in Figure 3a, no significant difference was found in pathological stage I LUAD patients harboring *BRAF* V600E mutation and without carrying *BRAF* V600 mutation (HR, 1.844, *p* = 0.226). After PSM and OW, similar results are presented in Figure 3b,c (adjusted HR, 1.144; *p* = 0.83 for PSM and adjusted HR, 1.466; *p* = 0.450 for OW, respectively). Cox regression was also performed to evaluate the prognostic role of *BRAF* V600E mutation. The results are shown in Table S2. In the univariable analysis, *BRAF* V600E mutation status was not significantly associated with RFS (HR, 1.844; *p* = 0.226). Only gender (HR, 0.747; *p* = 0.045), predominant pattern (all *p* < 0.01), tumor size (all *p* < 0.05), VPI (HR, 3.846; *p* < 0.001), LVI (HR, 5.958; *p* < 0.001) and ACT (HR, 1.563; *p* = 0.003) were significantly associated with RFS in the Cox regression analysis.

### Concurrent oncogenic driver mutations

**FIGURE 4:**
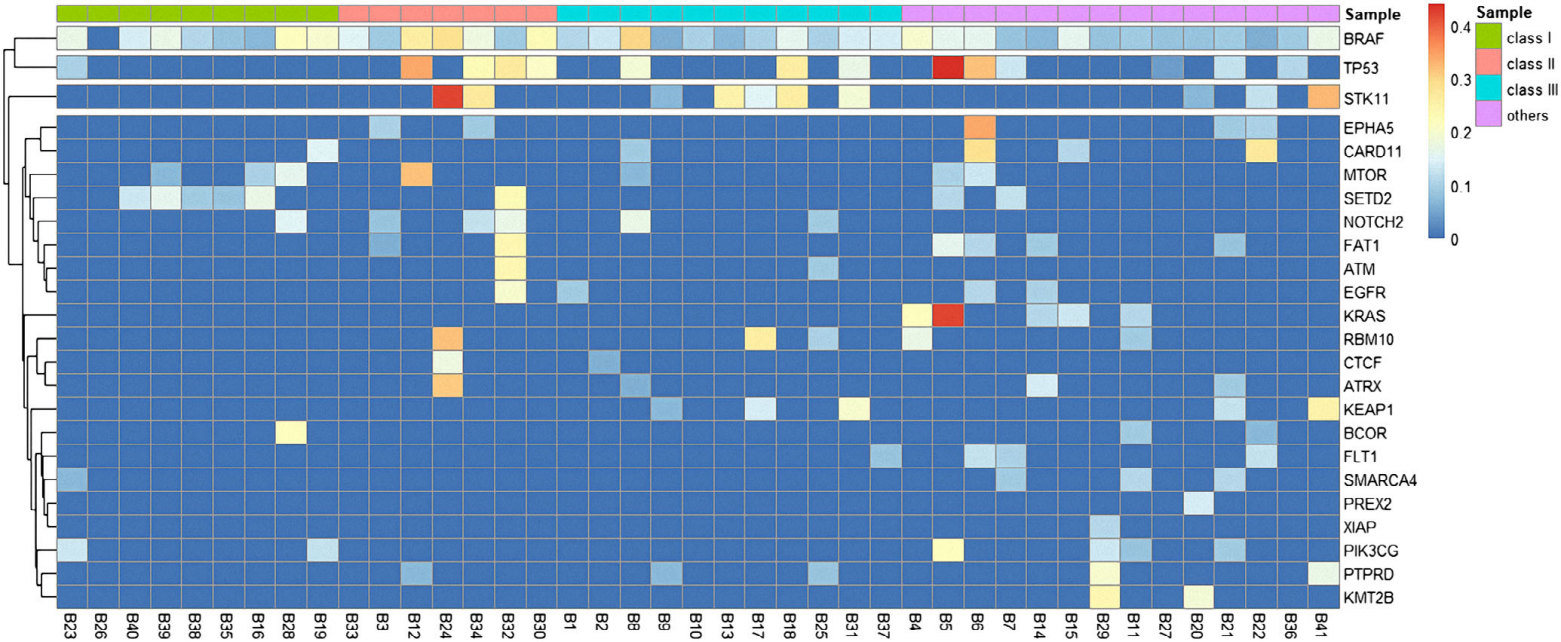
Mutation spectrum based on BRAF mutation class in 41 ADC patients.

*BRAF* mutations included four groups, class 1 (V600 E/K/D/R), class 2 (K601, L597, G464, and G469), class 3 (G466, N581, D594, and D596), and class 4 (others). Next, we assessed the concurrent oncogenic driver mutations with *BRAF* mutations in cohort 1. Collectively, 41 patients were found to harbor *BRAF* mutations in the initial 604 patients, in which class 1 accounted for 22.0%, class 2 for 17.1%, class 3 for 26.8%, and others for 34.1% (Table S3). Most class 1 *BRAF* mutations were V600E mutations, with only one patient harboring the V600K mutation. The most frequent concurrent mutation was TP53 (15, 36.6%), followed by STK11 (10, 24.4%), SETD2 (8, 19.5%), MTOR (7, 17.1%), EPHA5 (6, 14.6%), FAT1 (6, 14.6%), and NOTCH2 (6, 14.6%) (Figure 4). Of note, 230 (38.1%) patients were found to harbor *KRAS* mutations while in the *BRAF*‐mutant subgroup, only five *KRAS* mutations were observed and they all belonged to the non‐V600 class, which may indicate that class 1 mutations were mutually exclusive from *KRAS* mutations. STK11 and EPHA5 mutations were also less likely to occur in *BRAF* class 1 mutations. Four patients carrying non‐V600 *BRAF* mutations were found to concurrently harbor *EGFR* mutations. We also attempted to evaluate the prognostic role of concurrent *BRAF* with TP53 or STK11 mutations. However, no significant difference was observed (Figure S1).

## DISCUSSION

This study aimed to evaluate the prognostic significance of *BRAF* mutations in patients with pathological stage I lung adenocarcinoma. The more we understand the clinical features and prognostic role of *BRAF* mutations, the more patients can be selected to undergo mutational screening and receive the appropriate therapeutic schedule. In cohort 1, the prevalence of *BRAF* V600E mutations was around 1.4%, while in cohort 2, the *BRAF* V600E mutation rate was estimated at 1.0%. The inconformity could be caused by ethnic differences, as the rate of *BRAF* mutations has been reported to be higher in Caucasian than in Asian populations.^17^, ^18^ The estimated *BRAF* mutation rate in lung adenocarcinoma was 5.6%, which is in line with previous studies.^3^, ^6^, ^18^, ^19^

Former studies revealed that NSCLC patients harboring *BRAF* V600E mutations occurred predominantly in females with a never‐smoking history, while non‐V600 mutations were mainly found in male smokers.^17^, ^20^, ^21^ In cohort 2, most patients (93.8%) harboring *BRAF* V600E mutation never had a smoking history. In contrast, most patients (83.3%) harboring *BRAF* mutations in cohort 1 had a smoking history. The uneven distribution of *BRAF* mutation types between two cohorts may partly explain the reason. The predominant histological pattern of BRAF‐mutant tumors in cohort 1 was acinar/papillary (54.2%), followed by micropapillary/solid (20.8%). When focusing on the subgroup of class 1 mutations, the proportion of micropapillary/solid accounts for 33.3% (2 in 6 patients), which was consistent with previous reports that *BRAF* V600E‐mutant tumors were more likely to show a histological type characterized by micropapillary features.^20^ In our cohort 2, a high proportion of VPI (18.8%) in the *BRAF* V600E mutation group was also observed. Limited by the small sample size, statistical significance was not reached. Dexter et al. also reported that the odds of group I mutations were higher (odds ratio:4.39, 95% CI:1.11–17.4) among tumors involving the pleural space.^22^ Nevertheless, the mechanism behind is of interest and needs to be further investigated.

It has been previously reported that BRAF V600E mutations are generally mutually exclusive from ALK rearrangements and *EGFR* mutations.^2^, ^19^ In our study, we found two patients with concomitant *EGFR* and *BRAF* V600E mutation, one with EGFR 19‐Del and one with *EGFR*‐L858R mutation in cohort 1. In cohort 2, two patients harboring concomitant *EGFR* and *BRAF* mutations were also observed, one with *EGFR* V292L mutation, and one with *EGFR* X901_splice mutation. However, these two patients belonged to the non‐V600E *BRAF* mutation class. Li et al. reported five patients in the series with concurrent *BRAF* V600E plus *EGFR* mutations.^23^ Liza et al. also reported a 16% rate of double mutation among patients carrying *BRAF* mutations.^24^ Indeed, recent studies demonstrated that due to the intrinsic heterogeneity of intratumor, EGFR‐mutated NSCLC could seldom carry additional *BRAF* mutations, which is considered a predictor of resistance to EGFR inhibitors and tumor rapid progress.^25^, ^26^ In addition, Kinno et al. pointed out that tumors with V600E *BRAF* mutations were mutually exclusive from *KRAS* mutations.^17^ Concurrent *BRAF* V600E plus *KRAS* mutations were also not found in this study, indicating such mutation combinations may be rarer than others.

In some other types of tumors, such as melanoma, papillary thyroid carcinoma and colorectal cancer, the association between *BRAF* mutation and poor survival has been documented.^27^, ^28^ However, the prognostic impact of *BRAF* mutation in NSCLC patients is sustained by scarce evidence and existing studies are often contradictory. Xi et al. analyzed 1680 patients with NSCLC and identified 28 patients harboring *BRAF* mutation. Patients with V600E‐mutated tumors had a similar PFS to first‐line chemotherapy compared to patients with non‐V600E mutations (5.2 vs. 6.4 months; *p* = 0.561).^18^ Cardarella et al. screened 883 tumors and found advanced NSCLC patients with *BRAF* mutations and wild tumors had no difference in OS and showed similar PFS to platinum‐based chemotherapy. Nevertheless, Marchetti et al. demonstrated that patients with V600E‐mutated NSCLC had significantly shorter disease‐free and overall survival rates (HR, 2.19; *p* = 0.11 and HR, 2.18; *p* = 0.014, respectively) in a retrospective study which included 1046 NSCLC patients.^20^

In our study, we first analyzed 431 patients with radical resection LUAD and found 24 patients harboring BRAF mutations. Before and after PSM and OW, *BRAF* mutation patients showed a similar RFS trend compared with *BRAF* wild‐type patients. Cox regression analysis also excluded *BRAF* mutations as an independent factor to predict RFS. Next, we further investigated the prognostic role of *BRAF* V600E mutation, and RFS was not significantly different between *BRAF* V600E mutation and without *BRAF* V600E mutation patients. Taken together, we conclude that the BRAF mutation status lacks prognostic significance in stage I lung adenocarcinoma patients.

There were some limitations in our study. First, despite two propensity‐score methods being utilized in our study, our analysis was still limited by its retrospective nature. Second, because of the low incidence of *BRAF* mutations, the sample size of *BRAF* mutations was relatively small. Further large multicentric studies are required to research and verify our results. Third, the follow‐up time was relatively short, and we will continue to observe these patients. Fourth, the genotype of BRAF in cohort 2 was detected by ARMS‐PCR. Although ARMS‐PCR can detect *BRAF* V600E mutations in NSCLC patients with high sensitivity, non‐V600E *BRAF* mutations can hardly be detected. Limited by the technology, we could only analyze the prognostic role of V600E *BRAF* mutations in cohort 2, losing sight of class II and class III *BRAF* mutations.

In conclusion, in this study, we demonstrated that BRAF status may not be capable of predicting prognosis in this population. Further studies are required to confirm our findings.

## AUTHOR CONTRIBUTIONS

Conception and design: Dong Xie, Shang‐Shang Ma, and Lei‐Lei Wu. Administrative support: Dong Xie. Provision of study materials or patients: Dong Xie, Shang‐Shang Ma, Kun Li, Rang Rang Wang, and Le‐Lei Wu. Collection and assembly of data: Shang‐Shang Ma, Jia‐Yi Qian, Zhi‐Ye Huang, Yu'e Liu, and Ming‐Jun Li. Data analysis and interpretation: Dong Xie, Shang‐Shang Ma, Ming‐Jun Li, Zhi‐Ye Huang, Yu'e Liu, Rang‐Rang Wang, and Lei‐Lei Wu. Manuscript writing: All authors. Final approval of manuscript: All authors.

## CONFLICT OF INTEREST STATEMENT

The authors declare no competing interests.

## Supporting information


**FIGURE S1.** Kaplan–Meier survival curves for patients with concurrent BRAF with TP53 mutations (a) and STK11 mutations (b).

**TABLE S1.** The baseline characteristics of patients in cohort 2.

**TABLE S2.** Univariable survival analyses of patients in cohort 2 for RFS.

**TABLE S3.** BRAF mutation classes included in cohort 1.

## Data Availability

The data sets are available from the corresponding author upon reasonable request.
