# Supplementary figures and images for: Is there a prognostic difference among stage I lung adenocarcinoma patients with different BRAF ‐mutation status?
Source: Thorac Cancer. 2024 Feb 16;15(9):715–21. doi: 10.1111/1759-7714.15248 (PMC10961218; doi:10.1111/1759-7714.15248)

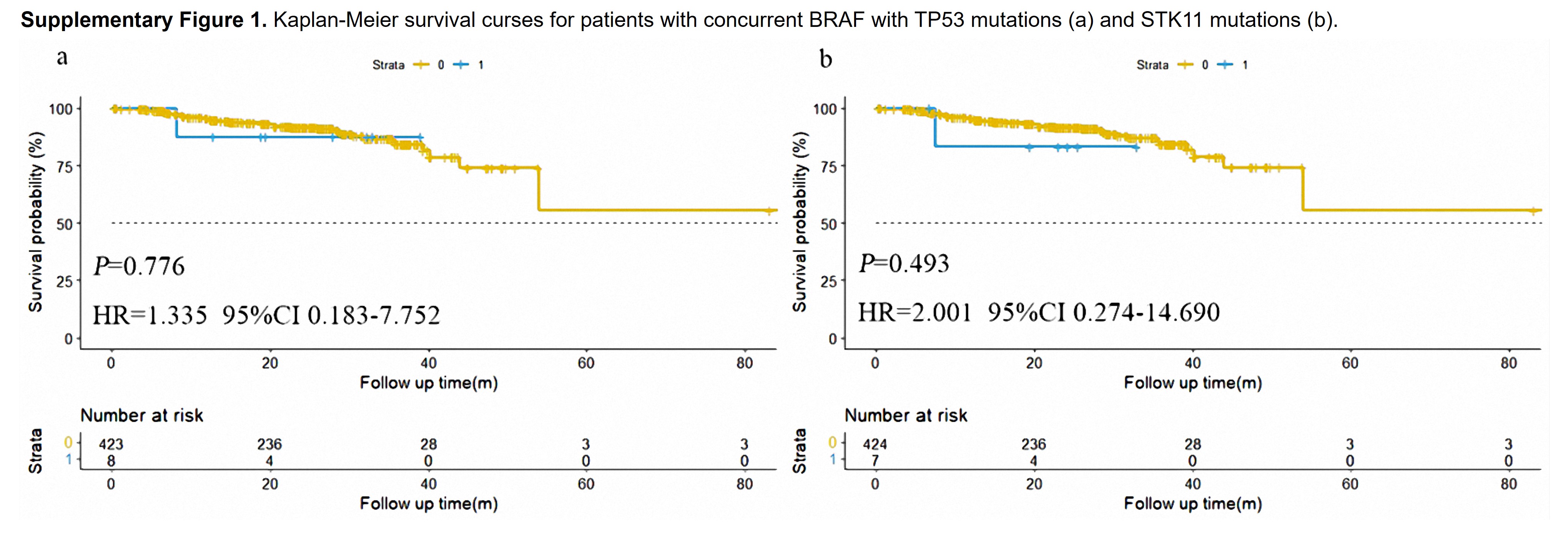

Supplement: Supplementary file 1 — FIGURE S1. Kaplan–Meier survival curves for patients with concurrent BRAF with TP53 mutations (a) and STK11 mutations (b). [file TCA-15-715-s004.jpg]
