# Supplementary material for: Is there a prognostic difference among stage I lung adenocarcinoma patients with different BRAF ‐mutation status?
Source: Thorac Cancer. 2024 Feb 16;15(9):715–21. doi: 10.1111/1759-7714.15248 (PMC10961218; doi:10.1111/1759-7714.15248)
Supplement: Supplementary file 2 — TABLE S1. The baseline characteristics of patients in cohort 2. [file TCA-15-715-s003.docx]

**Supplementary Table 1. The baseline characteristics of patients in cohort 2.**

| **Variables** | **Without V600E mutation** | **V600E-mutant** | ***P* -value** | **SMD** |
| --- | --- | --- | --- | --- |
| Gender |  |  | 0.704 | 0.158 |
| Male | 919(57.9%) | 8(50.0%) |  |  |
| Female | 669(42.1%) | 8(50.0%) |  |  |
| Age at surgery, years |  |  | 1.000 | 0.008 |
| ≤65 | 999(62.9%) | 10(62.5%) |  |  |
| >65 | 589(37.1%) | 6(37.5%) |  |  |
| Smoking history |  |  | 0.429 | 0.335 |
| No | 1321(83.2%) | 15(93.8%) |  |  |
| Yes | 267(16.8%) | 1(6.2%) |  |  |
| Extent of surgery |  |  | 0.935 | 0.102 |
| Lobectomy | 1448(91.2%) | 15(93.8%) |  |  |
| Sub-lobectomy | 140(8.8%) | 1(6.2%) |  |  |
| Predominant pattern |  |  | 0.147 | 0.618 |
| Lepidic | 570(35.9%) | 3(18.8%) |  |  |
| Acinar/Papillary | 850(53.5%) | 11(68.8%) |  |  |
| Micropapillary /Solid | 99(6.2%) | 0(0.0%) |  |  |
| Others | 69(4.3%) | 2(12.5%) |  |  |
| Tumor size, cm |  |  | 0.908 | 0.189 |
| <1 | 155(9.8%) | 2(12.5%) |  |  |
| 1-2 | 756(47.6%) | 8(50.0%) |  |  |
| 2-3 | 521(32.8%) | 4(25.0%) |  |  |
| 3-4 | 156(9.8%) | 2(12.5%) |  |  |
| VPI |  |  | 0.594 | 0.209 |
| Absent | 1408(88.7%) | 13(81.2%) |  |  |
| Present | 180(11.3%) | 3(18.8%) |  |  |
| LVI |  |  | 1.000 | 0.147 |
| Absent | 1571(98.9%) | 16(100.0%) |  |  |
| Present | 17(1.1%) | 0(0.0%) |  |  |
| STAS |  |  | 0.842 | 0.194 |
| Absent | 1551(97.7%) | 15(93.8%) |  |  |
| Present | 37(2.3%) | 1(6.2%) |  |  |
| ACT |  |  | 0.877 | 0.107 |
| No | 1115(70.2%) | 12(75.0%) |  |  |
| Yes | 473(29.8%) | 4(25.0%) |  |  |
| Stage |  |  |  |  |
| IA1 | 153(9.6) | 1(6.2) | 0.960 | 0.145 |
| IA2 | 706(44.5) | 7(43.8) |  |  |
| IA3 | 428(27.0) | 5(31.2) |  |  |
| IB | 301(19.0) | 3(18.8) |  |  |

SMD, standardized mean difference; VPI, Visceral pleural invasion; LVI, Lymphovascular invasion; STAS, Spread through air spaces; ACT, adjuvant chemotherapy.
