# Supplementary material for: Is there a prognostic difference among stage I lung adenocarcinoma patients with different BRAF ‐mutation status?
Source: Thorac Cancer. 2024 Feb 16;15(9):715–21. doi: 10.1111/1759-7714.15248 (PMC10961218; doi:10.1111/1759-7714.15248)
Supplement: Supplementary file 3 — TABLE S2. Univariable survival analyses of patients in cohort 2 for RFS. [file TCA-15-715-s002.docx]

**Supplementary Table 2. Univariable survival analyses of patients in cohort 2 for RFS.**

|  | **Univariable analysis** | | |
| --- | --- | --- | --- |
| **Variables** | **HR** | **95%Cl** | ***P-*value** |
| Gender |  |  |  |
| Male | 1 |  |  |
| Female | 0.747 | 0.562-0.994 | 0.045 |
| Age at surgery, years |  |  |  |
| ≤65 | 1 |  |  |
| >65 | 1.893 | 1.424 | 2.517 |
| Smoking history |  |  |  |
| No | 1 |  |  |
| Yes | 1.414 | 0.994-2.011 | 0.054 |
| Extent of surgery |  |  |  |
| Lobectomy | 1 |  |  |
| Sub-lobectomy | 0.813 | 0.459-1.441 | 0.479 |
| Predominant pattern |  |  |  |
| Lepidic | 1 |  |  |
| Acinar/Papillary | 3.011 | 2.006-4.521 | <0.001 |
| Micropapillary /Solid | 7.689 | 4.612-12.820 | <0.001 |
| Others | 3.312 | 1.616-6.785 | 0.001 |
| Tumor size, cm |  |  |  |
| <1 | 1 |  |  |
| 1-2 | 2.695 | 1.161-6.256 | 0.021 |
| 2-3 | 6.072 | 2.637-13.982 | <0.001 |
| 3-4 | 5.122 | 2.074-12.650 | <0.001 |
| VPI |  |  |  |
| Absent | 1 |  |  |
| Present | 3.846 | 2.821-5.244 | <0.001 |
| LVI |  |  |  |
| Absent | 1 |  |  |
| Present | 5.958 | 3.047-11.649 | <0.001 |
| STAS |  |  |  |
| Absent | 1 |  |  |
| Present | 1.471 | 0.652-3.319 | 0.352 |
| BRAF V600E mutation |  |  |  |
| No | 1 |  |  |
| Yes | 1.844 | 0.684-4.970 | 0.226 |
| ACT |  |  |  |
| No | 1 |  |  |
| Yes | 1.563 | 1.166-2.093 | 0.003 |

RFS, Recurrence-free survival; HR, hazard ratio; CI, confidence interval; VPI, Visceral pleural invasion; LVI, Lymphovascular invasion; STAS, Spread through air spaces; ACT, Adjuvant chemotherapy.
