# Supplementary material for: Is there a prognostic difference among stage I lung adenocarcinoma patients with different BRAF ‐mutation status?
Source: Thorac Cancer. 2024 Feb 16;15(9):715–21. doi: 10.1111/1759-7714.15248 (PMC10961218; doi:10.1111/1759-7714.15248)
Supplement: Supplementary file 4 — TABLE S3. BRAF mutation classes included in cohort 1. [file TCA-15-715-s001.docx]

**Supplementary Table 3. BRAF mutation classes included in cohort 1.**

| **BRAF mutations** | **Frequency** |
| --- | --- |
| Class I |  |
| V600 E/K | 9 |
| Class II |  |
| K601 N/E | 2 |
| G469 A/V | 3 |
| E695 Q | 1 |
| T599 I | 1 |
| Class III |  |
| G466A/V | 3 |
| D594N/G | 3 |
| N581 S/I | 3 |
| G596 R | 1 |
| V459 L | 1 |
| Others | 14 |
